# Innovating Care in Multiple Sclerosis: Feasibility of Synchronous Internet-Based Teleconsultation for Longitudinal Clinical Monitoring

**DOI:** 10.3390/jpm12030433

**Published:** 2022-03-10

**Authors:** Nima Sadeghi, Piet Eelen, Guy Nagels, Corinne Cuvelier, Katinka Van Gils, Marie B. D’hooghe, Jeroen Van Schependom, Miguel D’haeseleer

**Affiliations:** 1 Department of Neurology, Universitair Ziekenhuis Brussel (UZ Brussel), Laarbeeklaan 101, 1090 Brussels, Belgium; nima.sadeghi@uzbrussel.be (N.S.); guy.nagels@uzbrussel.be (G.N.); marie.dhooghe@mscenter.be (M.B.D.); 2 Nationaal Multiple Sclerose Centrum, Vanheylenstraat 16, 1820 Melsbroek, Belgium; piet.eelen@mscenter.be (P.E.); corinne.cuvelier@mscenter.be (C.C.); katinka.vangils@mscenter.be (K.V.G.); 3 Center for Neurosciences (C4N), NEUR and AIMS, Vrije Universiteit Brussel (VUB), Laarbeeklaan 103, 1090 Brussels, Belgium; jeroen.van.schependom@vub.be; 4 Icometrix, Kolonel Begaultlaan 1b, 3012 Leuven, Belgium; 5 Zebra Academy, Researchdreef 12, 1070 Brussels, Belgium; 6 Department of Electronics and Informatics (ETRO), Vrije Universiteit Brussel (VUB), Pleinlaan 2, 1050 Brussels, Belgium

**Keywords:** multiple sclerosis, teleconsultation, internet, feasibility, digital health

## Abstract

The ‘coronavirus disease of 2019’ crisis has recently forced an expedited adoption of teleconsultation (TC) in most medical domains. Short-term digital interventions have generally been associated with feasibility, clinical benefits, user satisfaction, and cost-effectiveness in patients with multiple sclerosis (MS) but outcomes after repeated utilization over extended periods need to be further evaluated. In this feasibility study, 60 subjects with MS were 1:1 randomized to receive standard care augmented by four TCs using an audiovisual Internet platform (intervention) versus standard care alone (controls), over a period of 12 months. Effects on functional status, medical costs, and satisfaction were explored as secondary outcomes. Eighty-nine out of 108 scheduled TCs (82.4%) were completed, and 26 patients could complete at least one TC (86.7%), meeting our prespecified feasibility target of 80%. The intervention did not lead to significant differences in functional status (with the potential exception of fatigue) nor medical costs. Most interventional patients declared themselves to be (very) satisfied about the quality of care and technical aspects associated with the TCs. Our results demonstrate that longitudinal clinical monitoring using real-time audiovisual TC over the Internet is feasible and well-received by patients with MS. Such an approach can be a promising new care strategy.

## 1. Introduction

Modern society, including the way we practice medicine, has been shaped by the technological progress inherited from the three industrial revolutions that have taken place since the mid-eighteenth century. An extension of the third phase is currently unfolding, characterized by a shift towards digital electronics, augmenting computing power, artificial intelligence, and a dominant role for the Internet. Telemedicine (TM)—sometimes also referred to as e-health or digital medicine—can be defined as the exchange of medical information between patients and healthcare providers, who are in separate locations, by means of electronic communication technology [1]. Phone calls and video conferencing are prototypical examples of directly interactive sessions in real-time, whereas their asynchronous counterparts (e.g., texting, email, self-scoring devices, wearable sensors, instructive tools) rely on store-and-forward transmission of medical data and/or advice [2,3]. TM applications have originally been developed to enable medical services that are difficult to deliver face-to-face, mainly due to time-sensitive requirements or geographic hurdles, but are now increasingly and more widely solicited as a complementary support in conjunction with the more traditional customs [4,5]. Especially in 2020, digital medicine has undergone an expedited adoption, as the ‘coronavirus disease of 2019’ (COVID-19) rapidly evolved into a global crisis, for which unprecedented social isolation and mobility restrictions have been installed as key measures of constrain [6,7], with teleconsultation (TC) becoming an essential escape scenario for a vast proportion of our health system [8,9,10].

Studies that have explored the impact of TM in patients with multiple sclerosis (MS), a frequently occurring, chronic inflammatory demyelinating and degenerative disorder of the central nervous system [11], were heterogeneous in terms of intervention type, methodology, and objectives (i.e., disability assessment, disease management, remote treatment, rehabilitation/exercise) but have generally shown that the applied procedures provide clinical benefit, user satisfaction, time gain, and/or cost-effectiveness [3,12]. Notably though, scientific data on synchronous communication for TC purposes remain scarce in this field. Two independent pilot studies have recently demonstrated that such single sessions, using an audiovisual Internet platform, were feasible and well-received in patients with MS [13,14]. The outcomes after repeated utilization over extended periods of time, however, are still unknown and form the subject of our current paper, hereby addressing an important and actual knowledge gap in the anticipation that TC—once we have reached the post-COVID-19 era—will consolidate its newly obtained place in the MS clinic [15].

## 2. Materials and Methods

### 2.1. Study Design, Objectives and Ethics

We performed a single-center prospective study exploring the feasibility (primary endpoint) of multiple planned synchronous TCs, using an audiovisual Internet platform, in the longitudinal clinical monitoring of patients with MS. Effects on functional status, medical costs estimates, and satisfaction were assessed as secondary outcomes; a randomized control trial design was adopted because most of these exploratory analyses required a comparison to standard care. Our study was approved by the ethics committee of the Nationaal Multiple Sclerose Centrum (NMSC) Melsbroek (local; internal reference: AvN/AVDZ) and the Universitair Ziekenhuis Brussel (leading; internal reference: 2018/269, Belgian Unique Number: 143201836797). Written informed consent was obtained from all participants prior to inclusion. Recruitment and teleconsulting procedures were similar to a preparatory pilot, in which one digital visit was planned in twenty patients with MS [14], who all agreed to be involved in this longitudinal project as well.

### 2.2. Participants

Sixty French- and/or Dutch-speaking patients with MS, according to the 2017 revised McDonald criteria [16], were recruited at the NMSC Melsbroek, which is a specialized Belgian MS center, during routine medical follow-up. Home access to the Internet with a webcam-equipped device was mandatory for study participation. Inclusion of individuals with a high suspicion of moderate to severe cognitive impairment (based on common sense judgement of the medical record and/or initial patient contact; any known cognitive dysfunction defined as scoring less than 21 on the Mini Mental State Evaluation, if present in the medical record, was used as an exclusion criteria) was actively avoided. Eligible candidates were 1:1 randomized to receive standard care (control group) versus standard care, augmented by four scheduled TM visits (intervention group) over the following 12 months, using “Research Randomizer” (https://randomizer.org; last accessed on 22 February 2020).

### 2.3. Teleconsultations

The study period started with an inclusion visit and terminated with a close-out evaluation (both face-to-face), conducted by the study supervisor (MiD), with an interval of 12 ± 2 months between both. Subjects in the intervention group received the date and time of their first TC after randomization, and subsequent appointments were planned at the end of each digital contact, striving for an equal distribution over the study period (i.e., one TC every 3 months). The rescheduling of digital appointments was permitted as this regularly occurs with standard visits as well. All TCs were performed by the same MS nurse or neurologist—derived from a pool of three consulting nurses (PE, CC, KVG) and one neurologist (MiD)—for each individual patient, using an Internet-based audiovisual communication platform obtained from Zebra Academy [17]. The main goal of the TCs was to explore the patient’s current global health status during a routine clinical consult. A checklist (with questions about e.g., general and neurological health, medication, and life-style factors—see Table in Ref. [14]), similar to the usual content of a regular face-to-face MS consultation, was used as the backbone for the conversation, but deviations at the patient’s initiative were allowed. Patients were provided with a unique hyperlink by email in advance, leading them directly to the virtual waiting room where they could see and accept our incoming call (see Figure in Ref. [14]). Access was possible from any device with a webcam (i.e., laptop, desktop, tablet, smartphone). Google Chrome (Google LLC, Mountain View, CA, USA) was used as the web browser on both sides of the connection, as advised by Zebra Academy. All subjects had 30 min to respond to the call, starting from the scheduled time, with maximum of three attempts. A written report was forwarded by the study team to the treating neurologist after each TC.

### 2.4. Evaluations

#### 2.4.1. Feasibility

Our TC approach was considered feasible if at least 80% of the patients in the intervention group could complete at least one digital visit and if at least 80% of the total number of scheduled digital visits could be completed (a priori defined).

#### 2.4.2. Functional Status

Clinical relapses during the study period were recorded, as self-reported by the patients, at the close-out visit. Disability, clustered symptomatology, and health-related quality of life were assessed at both the inclusion and close-out visit. The following variables were recorded: general disability with the Expanded Disability Status Scale (EDSS), mobility with the Timed 25-Foot Walk Test (T25FWT), dexterity with the Nine-Hole Peg Test (9HPT), information-processing speed with the Symbol Digit Modalities Test (SDMT), fatigue with the Fatigue Severity Scale (FSS), depression with the Beck Depression Inventory (BDI) and the Hospital Anxiety and Depression Scale (HADS), anxiety with the HADS, sleep quality with the Pittsburgh Sleep Quality Index (PSQI), and overall health-related quality of life with the Multiple Sclerosis Impact Scale (MSIS-29).

#### 2.4.3. Medical Costs

The number of emergency room visits, days of hospital admission (not including the study site) and visits to the general practitioner over the study period, as self-reported by the patients, were recorded at the close-out visit, as a proxy of medical costs.

#### 2.4.4. Satisfaction

The satisfaction with the study trajectory was enquired about for all patients, and their respective digital caregiver if applicable, at the close-out visit by means of 5-point Likert scales containing the following categories: very unsatisfied, unsatisfied, neutral, satisfied, very satisfied. Assessments were independently carried out for (a) global quality of care, (b) technical quality of the TCs, (c) convenience of the TCs, (d) quality of care of the TCs, and (e) added value of the TCs to medical care. Only item (a) was relevant for participants of the control group. Quantification per item was performed by giving a score of one for responding ‘very unsatisfied’ and increasing by one point for each higher response category.

### 2.5. COVID-19 Interference

**Figure 1:**
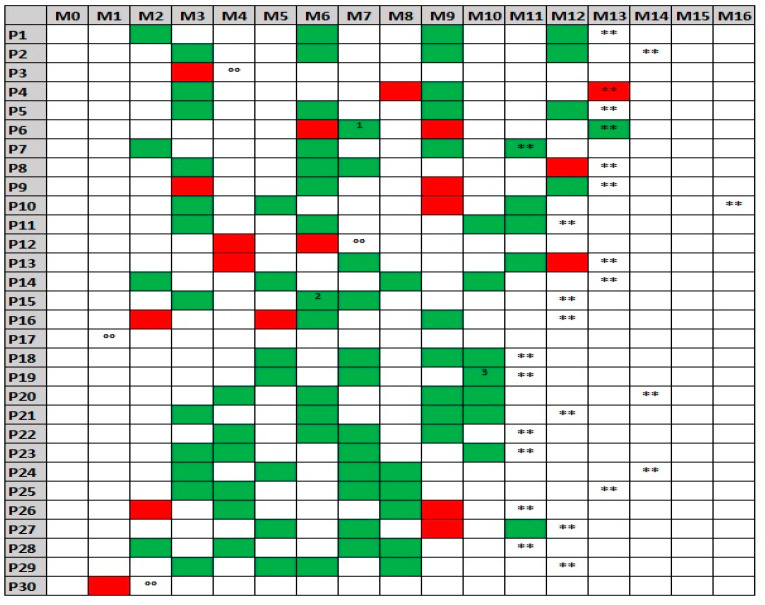
Timeline of study visits for each participant of the intervention group. M: month, P: patient. ** close out visit, °° drop-out. Successful teleconsultations are indicated in green, unsuccessful attempts in red. Corrections: 1 the successful teleconsultation was performed at M6, 2 two successful teleconsultations were performed at M6, 3 two successful teleconsultations were performed at M10.

Patients were enrolled in this project from 26 August 2019 to 21 February 2020. Due to unexpected COVID-19 measures, non-urgent medical appointments had to be postponed in Belgium on multiple occasions during the course of the study (particularly March–June and October–November 2020). This resulted in (a) two close-out evaluations falling outside the foreseen time frame (15 and 16 months after the inclusion visit, respectively) and (b) the need for scheduling several TCs sooner than initially intended. Timelines containing inclusion, close-out and all TC visits for each participant of the intervention group are displayed in the Figure 1.

### 2.6. Statistics

All statistical procedures were performed with GraphPad Prism version 9.0.0 (GraphPad Software; San Diego, CA, USA). Data are expressed as mean ± SD for reasons of uniformity. Group differences were assessed by means of unpaired Student *t* tests or Mann–Whitney *U* tests, where appropriate, depending on the data distribution revealed by Shapiro–Wilk testing. All reported *p* values are two-tailed and were considered statistically significant at the 0.05 level.

### 2.7. Data Availability

Anonymized data will be shared upon reasonable request from any qualified investigator.

## 3. Results

### 3.1. Participants

Baseline demographical data of all participants are shown in Table 1. Twenty-four of the patients randomized to the intervention group were under disease-modifying treatment at study inclusion (interferon beta: 1; teriflunomide: 1; dimethylfumarate: 3; fingolimod: 1; ocrelizumab: 4; natalizumab: 8; alemtuzumab: 6), versus 26 of the controls (interferon beta: 5; glatiramere acetate: 4; teriflunomide: 3; dimethylfumarate: 3; fingolimod: 1; ocrelizumab: 4; natalizumab: 6). Four subjects in each group dropped out during the study; reasons were loss of follow-up (one intervention subject who cancelled his first TC versus three controls), loss of interest (two intervention subjects versus one control), and a suitable device being no longer available (one intervention subject). Timing of drop-out in the intervention group can be deduced from the Figure 1.

### 3.2. Feasibility

Eighty-nine out of 108 scheduled TCs (82.4%) were successfully completed during the study while 26 patients could successfully complete at least one TC (86.7%). Failures were due to patients not responding (14/19) and technical issues (5/19). The non-responders were contacted at a later time by telephone and advised us that they either had forgotten the appointment or did not want to participate any longer (two subjects). Technical issues included no notification of the incoming call (three occasions), difficulties maintaining the Internet connection (one occasion), and insufficient quality of sound (one occasion). Isolated success rates were 21/29 (72.4%) for TC-1, 24/27 (88.9%) for TC-2, and 22/26 (84.6%) for TC-3 and -4.

### 3.3. Functional Status

The mean number of patient-reported relapses during the study period did not differ between participants of the intervention and control groups (0.3 ± 0.5 each, *p* = 0.54) who completed the study. Results of all other functional outcome measures are shown in Table 2 and Table 3. Mean changes were not statistically significant except for the FSS scores, which decreased by 0.3 ± 1.2 in patients of the intervention group, compared with an increase of 0.4 ± 1.0 in those of the control group (*p* = 0.03).

### 3.4. Medical Costs

No significant differences were observed in the mean number of emergency room visits (0.3 ± 0.5 versus 0.2 ± 0.5, *p* = 0.91), days of hospital admission (0.7 ± 2.7 versus 2.7 ± 8.0, *p* = 0.18), and the number of visits to a general practitioner (3.1 ± 4.5 versus 2.1 ± 2.1, *p* = 0.77) over the study period between patients of the intervention and control groups, respectively, who completed the study.

### 3.5. Satisfaction

Quantified results of the satisfaction enquiry are demonstrated in Table 4. The proportion of patients in the intervention group—who completed the study—declaring themselves to be satisfied or highly satisfied was 26/26 for global quality of care, 19/26 for technical quality of the TCs, 24/26 for convenience of the TCs, 24/26 for quality of care of the TCs, and 23/26 for added value of the TCs to medical care; results for the health professionals who performed the TCs were 25/26, 16/26, 21/26, 25/26, and 25/26, respectively.

## 4. Discussion

We present the first ever study, to our knowledge, demonstrating the feasibility of synchronous TC using an audiovisual Internet platform, when repeatedly applied for the clinical monitoring of individuals with MS over a substantial time period (i.e., one year). In addition, the appraisal of general care, technical quality, and convenience, as related to the digital visits, was excellent in the majority of patients and healthcare providers involved in the interventional arm. These results are in line with a recent and similarly designed trial among subjects with Parkinson disease—representing another frequently occurring chronic disorder of the central nervous system—living throughout the United States of America, in which 98% of the 97 participants randomized to the intervention group completed at least one digital visit, and 91% of the 388 planned digital visits were completed as scheduled. Here, the authors calculated that each virtual session would have saved patients a median of 88 min (95% CI 70–120; *p* < 0.001) and 38 miles per visit (95% CI 36–56; *p* < 0.001), compared to their usual care [18]. Feasibility was considered as a binary outcome measure in both studies (i.e., either the TC works or not) and the prespecified success target of at least 80% corresponds well to previously reported ‘no-show’ rates associated with in-person visits at neurology clinics [19,20].

Our TC approach did not lead to significant differences in parameters reflecting functional status (with the exception of fatigue) and medical costs. These secondary analyses were incorporated mainly for exploratory purposes, accepting a possible risk of a type II error. The likelihood of inducing a clinically relevant benefit with our intervention was considered low during study preparation, since it was never the intention to offer specific nor standardized treatment programs. Nonetheless, retaining the status quo can also be valuable as we, a priori, did consider the possibility of (increased) virtual attention inducing or aggravating MS-related symptomatology, such as anxiety and/or sleep difficulties. The positive impact of the intervention on fatigue levels should be interpreted with caution because the effect size was small and would not have survived a statistical (Bonferoni) correction for multiple comparisons. It is worth noting, however, that fatigue and physical activity levels showed improvement with other web- and telephone-based interactive sessions primarily based on educational and motivational coaching [21,22]. Surrogates of medical costs did not significantly differ between our two groups and were preferred above direct values because TC still occurred in addition to standard care. Gain in that domain, though, can be expected in future studies which actually replace face-to-face with digital visits in routine follow-up.

COVID-19 has deeply disrupted human socialization and forced our health system towards a large-scale adoption of TC on very short notice. Our findings can help solidify the scientific basis for continuing such practice, using the Internet as the flagship of modern-day communication, within a complementary hybrid model for next-generation MS care [15]. A number of reasons can be given to explain why patients with MS may be particularly suitable candidates to benefit from digitalization of neurological care. First, diagnosis is typically established during young adulthood [11], when time availability for medical attention is limited due to other priorities in life. Second, MS may lead to cumulative physical disability [11], complicating access to neurological facilities, even in areas offering sufficient and dense resources. Third, over the past two decades, we have seen a spectacular growth of the disease-modifying and symptomatic treatment armamentarium for individuals with MS, which is expected to ameliorate at least short- to medium-term prognosis but also increases the complexity of routine follow-up. Fourth, a significant proportion of affected patients (i.e., 30%) appears to completely miss out on neurological care [23], likely decreasing their chance of receiving state-of-the-art disease management and jeopardizing a favorable clinical outcome. Simultaneously, there seems to be a high interest among individuals with MS for using the Internet as a health information source and for online interaction with medical specialists [24]. As a final, general, and perhaps most convincing argument, we can state that nervous system disorders are currently the leading source of disability, affecting over one billion people worldwide [25], and represent a burden that is expected to at least double over the next two decades, mostly because of a growing elderly population [26]. In parallel, we have recently witnessed an increased prevalence and life-expectancy in patients with MS [27,28]. Access to neurological care is indigent already, as expressed by several health authorities in statements that do not solely apply to remote or low-income regions [4]. Consequently, the pressure on our classic health model will naturally rise to levels necessitating at least some form of digital redesign in order to avoid a total overflow, a rationale that has recently been put to the forefront by COVID-19 but survives even in complete abstraction of this crisis.

The most important limitations of this study were caused by the heterogeneity of the healthcare provider pool and the absence of a clinical neurological exam in the TC protocol. Neurology has long remained a very bedside-orientated ‘hands-on’ specialty, and the fear of missing subtle yet critical clinical details during a remote physical evaluation likely forms the Achilles heel of teleneurology in general. Supportive proposals are starting to be published [3,29], and it is of interest to mention that Bove and colleagues recently reported an agreement within one point between in-person and televideo-enabled EDSS scores for 88% of the cases, which is similar to the in-person inter-rater reliability described earlier by others [30]. The NMSC Melsbroek is a highly specialized hospital specifically focusing on the neurological management, multidisciplinary care, and/or rehabilitation of individuals with MS. All participants were allowed to use the ambulatory and in-hospital rehabilitation services of the center as a part of standard care during the study, if deemed necessary by their treating physicians. This decision was based on ethical considerations, but it cannot be excluded that such rehabilitation activities have influenced our secondary outcomes. As explained above (see Section 3.5), a priori unforeseen COVID-19 measures may have resulted in reduced access to the clinic and/or delay of standard care on multiple occasions during the study, which could have created a disproportionately positive welcoming of digital solutions. Furthermore, there might be other reasons to be cautious when generalizing our findings to the full MS community, as it cannot be ruled out that disease-specific, geographic, cultural, and/or social differences may lead to less positive outcomes. Previous studies have disclosed that patients with cognitive or visual impairment experienced more difficulties while using home-based TM systems [31,32], whereas we have actively avoided recruitment of participants with apparent cognitive dysfunction. We also have to acknowledge that there was a greater proportion of participants with a higher education level in our interventional group, as compared to the controls. TM has generally been praised for its potential to facilitate access to medical care, but recent reports have, somewhat surprisingly, warned of the persistence or even aggravation of ethnical and other disparities [33,34,35]. Possible solutions include wide-spread promotional campaigns, mobile paramedical teams, assistance by (educated) caregivers, and fully equipped community centers, of which most examples can also help with the more challenging aspects of the clinical exam. These factors related to background variability should not be forgotten when designing future research aimed at demonstrating the non-inferiority of replacing face-to-face with digital visits in the MS clinic, compared to the classic approach.

In conclusion, our study demonstrated the technical and practical feasibility of live audiovisual TC over the Internet for routine neurological follow-up in patients with MS. The digital approach was well-appreciated by both participating patients and healthcare providers. Future trials can now be designed in which the effect (e.g., non-inferiority) of replacing classic face-to-face visits with such TM modalities can be assessed for multiple purposes. We believe that this will be the phase where their full potential will come to expression but also one in which we must factor in the abovementioned systemic pitfalls and tailor the interventions to the individual patient needs.

## Figures and Tables

**Table 1:** Baseline demographic of the study participants.

	Intervention Group	Control Group
Number of subjects	30	30
Age * [years]	41.3 (10.4)	45.9 (9.1)
Gender [Female/Male]	19/11	15/15
MS subtype [RR/SP/PP]	21/8/1	21/7/2
Disease duration * [years]	10.1 (7.1)	11.2 (6.4)
Education [ES/HS/HE]	0/8/22	3/15/12
Employment status [U/E/S/D/R]	1/14/0/14/1	0/10/1/19/0

MS: multiple sclerosis, RR: relapsing-remitting, SP: secondary progressive, PP: primary progressive, ES: elementary school, HS: high school, HE: higher education, U: unemployed, E: employed (active), S: sick leave (temporary), D: disability leave (permanent), R: retired. * Data expressed as mean (SD).

**Table 2:** Functional outcomes in patients randomized to the intervention group who completed the study (*N* = 26) *.

	Inclusion	Close-Out	Change
EDSS	4.2 (2.1) [0]	4.2 (2.3) [0]	0.1 (0.9) [0]; 0.82
T25FWT	15.6 (34.4) [0]	26.6 (53.1) [1]	10.8 (34.1) [1]; 0.75
9HPT-dom	25.6 (13.7) [0]	24.7 (9.9) [1]	−0.8 (11.8) [1]; 0.82
9HPT-ndom	34.4 (53.5) [0]	37.4 (55.0) [1]	2.4 (11.9) [1]; 0.77
SDMT	58.3 (12.5) [2]	55.0 (13.6) [2]	−1.8 (10.0) [4]; 0.38
FSS	4.9 (1.2) [0]	4.6 (1.7) [0]	−0.3 (1.2) [0]; 0.03
BDI	10.9 (7.2) [0]	11.5 (11.4) [0]	0.7 (6.9) [0]; 0.56
HADS-anx	6.7 (4.0) [0]	6.3 (4.9) [0]	−0.3 (5.3) [0]; 0.55
HADS-dep	5.0 (3.5) [0]	5.5 (5.2) [0]	0.5 (4.6) [0]; 0.58
PSQI	6.3 (3.7) [1]	6.8 (4.7) [5]	−0.2 (4.0) [5]; 0.69
MSIS-29-phy	29.1 (20.2) [1]	34.6 (24.8) [0]	4.8 (18.1) [1]; 0.33
MSIS-29-psy	29.2 (22.5) [3]	29.2 (24.2) [0]	1.3 (23.9) [3]; 0.79

EDSS: Expanded Disability Status Scale; T25FWT: Timed 25-Foot Walk Test; 9HPT-dom: Nine-Hole Peg Test for dominant hand; 9HPT-ndom: Nine-Hole Peg Test for non-dominant hand; SDMT: Symbol Digit Modalities Test; FSS: Fatigue Severity Scale; BDI: Beck Depression Inventory; HADS-anx: Hospital Anxiety and Depression Scale for anxiety; HADS-dep: Hospital Anxiety and Depression Scale for depression; PSQI: Pittsburgh Sleep Quality Index (PSQI); MSIS-29-psy: Multiple Sclerosis Impact Scale for psychological impact; MSIS-29-phy: Multiple Sclerosis Impact Scale for physical impact. * Scores expressed as mean (SD) [missing values]; *p* value for comparison with the respective change in the control group, as expressed in Table 3.

**Table 3:** Functional outcomes in patients randomized to the control group who completed the study (*N* = 26) *.

	Inclusion	Close-Out	Change
EDSS	4.4 (2.1) [0]	4.6 (2.0) [0]	0.2 (0.7) [0]
T25FWT	6.8 (2.9) [2]	27.9 (74.3) [0]	0.8 (2.2) [2]
9HPT-dom	48.9 (74.0) [0]	54.6 (77.9) [0]	5.6 (20.7) [0]
9HPT-ndom	50.0 (73.7) [0]	52.0 (74.4) [0]	2.1 (6.3) [0]
SDMT	53.6 (14.5) [4]	52.5 (13.9) [1]	0.9 (9.6) [5]
FSS	4.3 (1.2) [0]	4.6 (1.5) [0]	0.4 (1.0) [0]
BDI	8.8 (5.7) [0]	7.4 (4.9) [1]	−1.4 (3.8) [1]
HADS-anx	6.1 (3.6) [0]	6.4 (3.5) [1]	0.4 (3.7) [1]
HADS-dep	4.8 (3.1) [0]	4.5 (2.5) [1]	−0.2 (3.0) [1]
PSQI	6.4 (3.9) [0]	5.8 (3.4) [2]	−0.7 (3.4) [2]
MSIS-29-phy	33.1 (16.2) [3]	39.5 (18.2) [0]	7.7 (11.2) [3]
MSIS-29-psy	29.1 (22.0) [4]	29.8 (17.6) [0]	1.9 (17.6) [4]

EDSS: Expanded Disability Status Scale; T25FWT: Timed 25-Foot Walk Test; 9HPT-dom: Nine-Hole Peg Test for dominant hand; 9HPT-ndom: Nine-Hole Peg Test for non-dominant hand; SDMT: Symbol Digit Modalities Test; FSS: Fatigue Severity Scale; BDI: Beck Depression Inventory; HADS-anx: Hospital Anxiety and Depression Scale for anxiety; HADS-dep: Hospital Anxiety and Depression Scale for depression; PSQI: Pittsburgh Sleep Quality Index (PSQI); MSIS-29-psy: Multiple Sclerosis Impact Scale for psychological impact; MSIS-29-phy: Multiple Sclerosis Impact Scale for physical impact. * Scores expressed as mean (SD) [missing values].

**Table 4:** Satisfaction as quantified from 5-point Likert scales with regard to patients who completed the study *.

	Intervention Group (*N* = 26)	Control Group (*N* = 26)	HCPs Performing the TCs
Global QoC	4.6 (0.5) °	4.5 (0.5) °	4.4 (0.6)
Technical quality of the TCs	4.1(1.0)	-	3.9 (1.0)
Convenience of the TCs	4.5 (0.6)	-	4.2 (0.9)
QoC of the TCs	4.5 (0.6)	-	4.3 (0.7)
Added value of the TCs to medical care	4.4 (0.7)	-	4.6 (0.6)

HCPs: healthcare providers; TCs: teleconsultations; QoC: quality of care. * Scores expressed as mean (SD); ° *p* value not significant (0.58).

## Data Availability

Anonymized data will be shared upon reasonable request from any qualified investigator.
